# Endoscopic Palliation for Pancreatic Cancer

**DOI:** 10.3390/cancers3021947

**Published:** 2011-04-13

**Authors:** Mihir Bakhru, Bezawit Tekola, Michel Kahaleh

**Affiliations:** 1 Division of Gastroenterology and Hepatology, University of Virginia, PO Box 800708, Charlottesville, VA 22908, USA; E-Mail: mihirbakhru@gmail.com (M.B.); 2 Division of Medicine, University of Virginia, PO Box 800708, Charlottesville, VA 22908, USA; E-Mail: bdt2q@virginia.edu (B.T.)

**Keywords:** pancreatic adenocarcinoma, biliary obstruction, gastric outlet obstruction, endoscopy, stents, palliation

## Abstract

Pancreatic cancer is devastating due to its poor prognosis. Patients require a multidisciplinary approach to guide available options, mostly palliative because of advanced disease at presentation. Palliation including relief of biliary obstruction, gastric outlet obstruction, and cancer-related pain has become the focus in patients whose cancer is determined to be unresectable. Endoscopic stenting for biliary obstruction is an option for drainage to avoid the complications including jaundice, pruritus, infection, liver dysfunction and eventually failure. Enteral stents can relieve gastric obstruction and allow patients to resume oral intake. Pain is difficult to treat in cancer patients and endoscopic procedures such as pancreatic stenting and celiac plexus neurolysis can provide relief. The objective of endoscopic palliation is to primarily address symptoms as well improve quality of life.

## 1. Introduction

Pancreatic adenocarcinoma is the fourth leading cause of cancer-related mortality in the U.S. and seventh most common in Europe, due to vascular invasion or metastasis at the time of diagnosis [1-3].

The median survival is less than six months and only a minority (10–20%) of patients are considered candidates for resection at time of diagnosis. Accurate staging requires a multidisciplinary approach including imaging, tumor markers, and if available pathology via surgical exploration, to further classify the cancer's prognosis. Laparoscopic staging to determine resectability and thus prevent unnecessary exploratory laparotomies has had low detection rates and has fallen out of favor [4]. Even with those that are selected for surgical resection, the five year survival continues to be at 15–20%, primarily due to recurrence [5].

Given this poor prognosis, palliation may be the only option and is targeted at the multiple complications that can occur with pancreatic cancer. Improving symptoms, reducing hospitalization, and trying to enhance overall quality of life are amongst the goals of palliation. Palliation not only includes radiation or chemotherapy to reduce tumor burden but also surgical, radiologic, or endoscopic interventions [6-8].

Biliary obstruction, gastric outlet obstruction, and pain due to tumor advancement in adjacent nerve plexus or pancreatic duct involvement are common sequelae of cancer [6]. Endoscopic techniques have gained favor recently and considered first line treatment due to their lower complication rate and associated shorter hospital stay [9,10].

## 2. Management of Biliary Obstruction

Mass at the head of the pancreas is the most common location for pancreatic cancer, leading to biliary obstruction in 70–90% of patients [5,11]. Biliary obstruction can lead not only to jaundice, but pruritus, cholangitis, and hepatic dysfunction with eventual liver failure [6]. Patients survive longer if the obstructed biliary system is surgically bypassed [7]. Although surgery (preferably choledochojejunosotomy) can provide relief of obstruction and a chance of long term survival, the morbidity associated with it made endoscopic drainage more attractive [12-14]. In direct comparison to surgical drainage, endoscopic stent placement was noted to have lower procedure-related complications, improved quality of life, cost, and with similar mortality rate [9]. Several studies have analyzed cost implications of endoscopic versus surgical drainage for biliary obstruction. In one study, cost effectiveness of operative versus endoscopic management with stenting was compared for biliary obstruction from unresectable pancreatic cancer. Cost was higher from the surgical group driven by longer hospital stay and higher re-admission rate with equivalent duration of survival [15]. Artifon *et al.*, in a prospective randomized controlled trial, compared patients with metastatic pancreatic cancer with liver metastasis (n = 30) who underwent either endoscopic drainage (with metal stent) or surgical drainage to evaluate to cost and quality of life. The authors found less cost (p = 0.0013) and improved quality of life scores at 30 and 60 days (p = 0.042; p = 0.05) with endoscopic palliation. Interestingly, no difference was seen in rates of complications, re-admissions for complications or duration of survival [16].

Several studies have studied quality of life parameters in patients deemed unresectable and with biliary obstruction, and found that biliary stenting can improve their overall well-being [17]. In addition, other symptoms such as anorexia, fatigue, and insomnia can also improve with biliary drainage [18,19].

### 2.1. Endoscopic Technique

Prior to endoscopy, appropriate high resolution imaging (CT or Magnetic Resonance Imaging (MRI)) is typically obtained to delineate tumor burden and a reasonable roadmap of the biliary tree. Insertion of a biliary endoprosthesis is performed via endoscopic retrograde cholangiopancreatogram (ERCP) where selective cannulation of the bile duct achieved via a catheter and over a guidewire, a stent is typically advanced through a tight stricture, and deployed across the ampulla and into the duodenum. Biliary stent allows for drainage with subsequent relief of jaundice and pruritus with success rate of insertion at 90–95% in most experienced centers [20]. Endoscopic failures can occur and associated with limited access to major papilla from duodenal obstruction, inability to cannulate common bile duct or complex strictures. Complications include, but are not limited to, bleeding, cholangitis, pancreatitis and perforation.

### 2.2. Review of the Literature

Initially, plastic stents were used for palliation and rapidly gained popularity due to ease of use and relatively low cost. The median patency rates were approximately 3–4 months [21,22]. The main limiting factors to plastic stents not only included patency but also migration. In addition, the stent was a nidus for infection as intestinal bacterial flora had access to the biliary system, as well as obstruction due to a biofilm that eventually encases the stent lumen [23]. It was soon realized that larger diameter (then 10 French or 3.3 mm) would provide better stent patency [24,25].

With the evolution of self-expandable metal stents (SEMS) patency length was improved. Using small caliber catheter delivery system (7.5–10 French) accommodating the working channel of the endoscope, the SEMS is deployed across the malignant biliary stricture up to 30 French. The larger diameter allows for enhanced patency, up to 10 months. A meta-analysis comparing plastic to metal biliary stents demonstrated no statistical difference in technical success, therapeutic success, complications, or 30 day mortality but did show a lower risk of biliary obstruction for the latter [26]. Cost effectiveness in this analysis was inconclusive, thought to be due to patient length of survival. More recently, a study from Korea compared costs of metal versus plastic stent for unresectable malignant biliary obstruction, where costs of ERCP are lower than that of metal stents. Stent occlusion was higher in the plastic stent group in a shorter amount of time (133 *vs.* 278 days; p = 0.0004) as well as a higher rate of cholangitis and associated length of hospitalization, with no statistically significant cost difference ($1488.77 metal stent group *vs.* $1319.26 plastic stent group; p = 0.422) [27].

Interestingly and supported by the previous studies, survival was no different and fuels the debate about what stent is most appropriate. Most endoscopists would agree that prognosis plays a large part in this decision, as metal stents provide the most benefit in patients expected to survive at least 4–6 months [28]. The rationale for this approach is that if the patient will not survive for the next 4–6 months, the patency advantage will not be of benefit as plastic stents can be patent for 3–4 months [29-31].

SEMS initially were uncovered and this feature did not make them removable in case of malfunction and allowed for tumor ingrowth and mucosal hyperplasia [32]. These factors limited the patency and lead to the evolution of first partially covered and then fully covered SEMS, with the goal of negating tumor ingrowth. A multicenter randomized control trial comparing covered and uncovered SEMS showed better patency and lower occlusion for covered SEMS (mean 304 days *vs.* 166 days for uncovered stent) [33]. However more recent studies have failed to confirm this data [34,35]. Most recently, Telford *et al.* compared uncovered SEMS to partially covered SEMS (Wallstent, Boston Scientific, Natick MA) in patients with inoperable distal malignant obstruction and found no difference in rate of biliary obstruction (p = 0.53) or patient survival (p = 997), although the partially covered stent was prone to migration (p = 0061) [34]. It is still unclear which stent is ideal as limitations exist for both uncovered (described above) and for covered stents such as migration, and cholecystitis (by occluding cystic duct drainage-noted by both stent types) [36,37]. One new trend currently noticed in the literature is the increase use of metal stents in the setting of neo-adjuvant protocols (with delayed surgery) or as a first-intention intervention regardless of resectability as demonstrated by our group [36,38]. All data available so far seem to demonstrate the cost-effectiveness of such a concept [39]. Furthermore, a recent study by van der Gaag *et al.* argues against preoperative biliary decompression using plastic stents [40].

## 3. Management of Gastric Outlet Obstruction

Gastric outlet obstruction (GOO) is one of the later presentations of pancreatic cancer, occurring in 10–20% of patients, which leads to a mass effect from the pancreatic head obstructing the duodenum [41,42]. Historically, surgical bypass (via gastrojejunostomy) was the only option despite its significant morbidity and mortality. Recently, endoscopic approach to treat obstruction has emerged, while laparoscopic surgery remains another option [43].

### 3.1. Endoscopic Technique

In order to place a stent for relieving an obstruction, the location and the extent of tumor involvement is defined via imaging (*i.e.*, oral contrast study).

Endoscopic method gives better access to the duodenum with the ability to visualize to the site of obstruction. There are ongoing studies about the use of increasing types, shapes and sizes of stents with different compositions, however there are only two that are FDA approved: Enteral Wallstent (Boston Scientific, Natick, Massachusetts) and its newer version the Enteral Wallflex (Boston Scientific) for duodenal stenting that can be advanced into the working channel of a standard endoscope [44]. Once the site is reached, the selected stent should be 3–4 cm longer than the stricture, and may even require that stents be overlapped for adequate relief of obstruction [45]. Contrast and fluoroscopy is used to assure passage of obstruction as well as appropriate positioning of the stent. Common problems encountered with stenting of the duodenum include biliary obstruction in up to 40% of these patients requiring percutaneous or endoscopic drainage [46]. One proposal to avoiding this complication is to place a biliary stent before attempting enteral stenting. Failures are related to inability to cross the stricture due to complete obstruction, inaccessible distal strictures, functional gastric outlet obstruction, or peritoneal carcinomatosis or gastroparesis [45]. In these cases, the best option for palliation is to place a venting gastrostomy. As with endoscopic procedures, duodenal stenting carries risks of bowel perforation, bleeding, stent malposition and/or migration and worse case, fistula formation [45].

### 3.2. Review of the Literature

The success of duodenal stenting are measured by the patient returning to oral intake, with an average rate of 87–100% with minimal complication rates as low as 1.2% [47]. Philips *et al.* have one of the largest series of patients with enteral stenting involving 46 patients with reported 100% success rates of SEMS deployment and 91% clinical success rate [48]. Even though surgical bypass is historically a standard of treatment of GOO, recent data supports endoscopic duodenal stenting as a preferred alternative [9]. Stenting proved to be 1/3 of the cost of surgery with decreased hospital admission days [40]. Another major advantage is that patients are able to eat as early as two days post-operatively day as reported by Fiori *et al.*, which is a major goal of palliation [49].

## 4. Management of Pain

Pain is a debilitating symptom in pancreatic cancer which severely affects quality of life. Patients with advanced pancreatic cancer can present with severe pain due pancreatic duct obstruction or nerve involvement [50,51]. One way to alleviate the pain and improve quality of life is palliative pancreatic duct stenting to relieve the obstruction [50,52]. A recent review of available evidence shows complete relief of pain in 60% of patients and at least partial relief in 25% [53].

Although much of the data measures outcome in symptom control, there is paucity in data regarding the complication rates (including risks such as stent migration, occlusion, or infection) in these patients.

Since pain can be related to neural plexus involvement, celiac plexus ablation has been offered. The technique has evolved in the past century to currently include injection of neurolytic agents, such as phenol or alcohol, to treat the visceral afferent pain. Some have also suggested cryotherapy or even excision therapy [54]. The traditional posterior approach is fluoroscopy guided and usually performed by anesthesiologists. This method carried the rare risk of paralysis and paresthesia due to spinal cord injury [55].

Although not as well studied, several other approaches such as splanchnic neurolysis either via surgical or radiologic approach do exist but require further research of their success rates. Splanchnic neurolysis for instance appears to carry similar risk to celiac plexus neurolysis [56].

### 4.1. Endoscopic Technique

Celiac plexus block (CPB) was first described in 1914 by Kappis *et al.* and has since evolved to lead to development of celiac plexus neurolysis (CPN). Both methods are now used via endoscopic ultrasound (EUS) guidance for different purposes; where CPB is mostly used for chronic pancreatitis and CPN for pancreatic cancer pain [57]. The difference between CPB and CPN is in the injected agents and the location of injection. These procedures require that the endoscopist identify the antecrural space, noted to be anterior to the celiac plexus and inject the preferred agents in the appropriate location [58]. Once the appropriate landmarks are endoscopically identified, CPB involves injecting an anesthetic such as bupivacaine and a steroid in the area of the celiac plexus or on top of the ganglia [57]. CPN evolved with the increased recognition of the celiac ganglia via EUS. Levy *et al.* described the efficacy of direct injection into the ganglia [59]. This technique involves the same identification of structures; however, it requires directly injecting the celiac ganglia with a sclerosant, such as phenol or dehydrated alcohol with an anesthetic.

### 4.2. Review of the Literature

Multiple studies conclude that both CPB and CPN have a significant palliative role in chronic pancreatic abdominal pain. However, it is important to note that they do not eliminate the use of pre-procedure narcotic pain management. CPB appears to be most effective for chronic pancreatitis pain rather than pancreatic cancer pain as described by a recent meta-analysis [57]. The study concluded that there was an average of 51% reduction in abdominal pain. CPN on the other hand was more efficacious in pancreatic cancer pain, with an average of 73% reduction of pain. Levy *et al.* described EUS-guided CPN on patients with unresectable pancreatic carcinoma and chronic pancreatitis (n = 33). Ninety four percent (16/17) of patients with cancer and 4/5 with chronic pancreatitis reported pain relief. Interestingly, 34% had initial pain exacerbation thought to be related to improved therapeutic response (p < 0.05) [59]. A few studies reported that the efficacy of this procedure was dependent on various factors such as timing and location of the tumor. Mercadente *et al.* evaluated the use of narcotics post CPN and reported a transient reduction in supplemental narcotic pain management for up to four weeks post procedure then rebounded back to their original pain level if higher doses of opioids were note used. The authors concluded that CPN should be initiated earlier in the course of the pain to prevent the rebound, while the anatomy of the pancreas is still in tact to identify appropriate structures [60].

Catalano *et al.* reported the importance of tumor location in the efficacy of CPN, where patients with malignancy at the body or tail of the pancreas had better pain relief than those with pancreatic head cancer [61]. EUS-guided CPN carries well studied risks as described by Gunaratnam *et al.*, with hyperalgesia in 9%, postural hypotension in 20% and diarrhea in 7% of the patients, all of which were noted to be transient symptoms [62]. The benefit includes sustained pain relief for up to 24 weeks independent of adjuvant therapy [62].

## 5. Conclusions

Pancreatic cancer has very poor prognosis and often palliative procedures are the only option for patients to relieve their symptoms and improve their quality of life. Patients benefit from endoscopic approaches to treat biliary obstruction, gastric outlet obstruction, and cancer-related pain. Indication for endoscopic intervention is determined on a number of factors including patients' prognosis and life expectancy.
